# Nutritional factors associated with aggression

**DOI:** 10.3389/fpsyt.2023.1176061

**Published:** 2023-06-21

**Authors:** Olivia Choy

**Affiliations:** Department of Psychology, Nanyang Technological University, Singapore, Singapore

**Keywords:** nutrition, aggression, omega-3 (n-3), vitamin D, magnesium, zinc

## Abstract

Although the vast majority of patients in forensic psychiatry are treated using pharmacological agents, clinical and ethical concerns about their use have led to the consideration of alternative strategies to reduce aggression that is common in forensic psychiatric settings. One non-invasive and benign biologically-based treatment approach involves nutrition. This article provides a mini-review of the recent evidence on four salient nutritional factors associated with aggressive behavior, namely omega-3 fatty acids, vitamin D, magnesium, and zinc. The current evidence base indicates that lower omega-3 levels are associated with increased aggression. Although research on vitamin D and zinc in relation to aggressive behavior is more limited, there is initial evidence that they are negatively associated with aggression in healthy participants and in psychiatric samples. The relationship between magnesium and aggression varies depending on how magnesium is assessed. Findings from experimental trials reveal that nutritional intervention in the form of omega-3 supplementation has the potential to serve as an effective mode of treatment, with effects that can last beyond the intervention period. There is also support for the utility of nutrition to improve our understanding of how social processes are linked to aggression. In light of the nascent, but promising findings on the role of nutritional factors on aggressive behavior, directions for future research are discussed.

## Introduction

Aggression and violent incidents are common in psychiatric inpatient care. These largely comprise behaviors that physically threaten or harm people or property, although aggression can also be verbal in nature. One meta-analysis of 42 studies from 13 high-income countries revealed that 18% of patients committed violence in acute psychiatric units (1). Another review of 53 studies internationally showed that 23% of patients in psychiatric units exhibited aggression or agitation (2). Furthermore, across psychiatric facilities, the percentage of violent patients and rate of violent incidences were consistently highest in forensic psychiatric units (3, 4). These behaviors can have longer-term implications as increased assaults against staff are significantly associated with criminal recidivism after forensic psychiatric treatment (5).

To manage and reduce aggressive behavior, pharmacological treatments are commonly used in these settings. However, this is linked to some controversy. There are concerns about risks of diversion and dependence in a population with a high prevalence of substance use disorders and about potential differences in the efficacy of medications in different age groups (6). Additionally, although there is evidence of a small, statistically significant benefit of antipsychotics for aggression, given their significant side effects, it has been argued that their increased use may not be warranted, particularly in the presence of non-pharmacological treatments (7).

One alternative approach stems from the growing field of nutritional psychiatry. Forensic psychiatric inpatients have been found to have low average levels of omega-3 fatty acids and vitamin D, with nearly two-thirds of patients exhibiting inadequate vitamin D levels (8). Research also showed that in a jail, micronutrients such as omega-3, vitamin D, and magnesium did not meet nutritional intake guidelines, and only 3 days of a seven-day cycle menu met recommendations for zinc in men (9). To address these nutrient deficiencies, some randomized controlled trials (RCTs) have found broad-range supplements that comprise various vitamins, minerals, and macronutrients to be associated with reductions in aggressive and rule-breaking behavior in children with ADHD (10) and conduct problems (11), and in incarcerated samples (12–14). Others, however, reported that multivitamin, mineral, and omega-3 supplementation did not significantly reduce aggressive incidents in psychiatric inpatients (15) or in aggressive individuals with intellectual disabilities (16). The finding that very few studies examined the same composition of nutritional supplementation has resulted in a call for investigations of supplement content and dosage (17).

The aim of this mini-review is to summarize the current knowledge on salient nutritional components that have been proposed to be linked to aggressive behavior. Studies that examined omega-3 fatty acids, vitamin D, magnesium, and zinc in relation to aggression were searched on electronic databases (PubMed and Web of Science) until February 2023. Reference lists from articles were manually examined to find additional relevant articles. Following a review of the findings, remaining gaps in the literature are discussed with a view to inform the developing body of research on nutrition and aggression.

## Omega-3 fatty acids

Particular attention has been drawn to investigations of the role of omega-3 on aggressive behavior. Adding to meta-analytic evidence of a small, significant association between reduced omega-3 fatty acids and increased aggression in observational studies (18), data from a large nationwide birth cohort show that increased omega-3 intake by mothers during pregnancy was associated with fewer cases of aggressive behavior toward their baby (19) and that higher male dietary intake of omega-3 was associated with lower risk of physical violence toward their partners except in cases of extremely high omega-3 intake (20). A similar pattern of findings is seen at younger ages. Girls aged 9 to 13 years with aggressive behavior had significantly lower omega-3 intakes compared to non-aggressive girls (21). One contradictory finding that docosahexaenoic acid (DHA) and eicosapentaenoic acid (EPA), two important omega-3 fatty acids, were not correlated with aggression in violent schizophrenic patients may be attributed in part to the testing of omega-3 levels in plasma, rather than red blood cells which is preferred due to its lower biological variability (22).

Besides these observational studies, a meta-analysis and recent reviews on experimental studies have converged on the conclusion that there is initial support for the effectiveness of omega-3 supplementation to reduce aggression in offenders and in psychiatric contexts (18, 23). One observed trend is that the more “hot-blooded” reactive, impulsive aggression may be responsive to omega-3 interventions compared to more “cold-blooded,” planned, proactive aggression (24). Since these reviews, seven more RCTs involving omega-3 supplementation have been conducted. Supplementation significantly decreased self-reported physical aggressiveness in healthy adults (25) and parent and teacher-reported aggressive behavior in children with ADHD (26). Violent schizophrenic patients who took an omega-3 supplementation as an adjunct to antipsychotic medication for 4–12 weeks also showed significantly reduced aggression at a 12-week follow-up (27). This was not observed at weeks 4 and 8, suggesting that effectiveness may be tied to the duration of the intervention. Three experimental trials that assigned children to consume either more fish or more meat per week largely show no significant group differences in conduct problems (28–30). Findings from these studies may differ from those involving supplementation in the form of a drink or standard capsules because fish contains other nutrients and may substitute other foods in the diet which can counteract the effects of omega-3. These results may also be due to the lower oily fish intake, highlighting the need to consider treatment dosage. Taken together, studies on omega-3 supplementation have shown that significant treatment effects on aggressive behavior are observed in interventions that last from 6 weeks to 6 months, and with supplements containing 80 mg to 10.8 g of EPA and 116 mg to 5.4 g of DHA per day.

The duration of supplementation effects has largely not been examined. One exception is a study on young male offenders in prison who received omega-3 supplementation for 3 months. Compared with a placebo group and treatment-as-usual controls, they exhibited lower levels of antisocial behavior, particularly reactive aggression, up to 9 months after the end of the intervention (31), providing valuable insight about the efficacy of treatment using omega-3 for aggressive behavior in the longer term.

## Vitamin D

Vitamin D is a vitamin and prohormone as it can be ingested orally and produced endogenously in the skin by ultraviolet B conversion. In healthy community samples, observational studies have found low vitamin D status measured from dietary intake reports and blood samples to be linked to more aggression in adolescent girls (21, 32). Compared to children with lower vitamin D, those with higher vitamin D status based on serum 25-hydroxyvitamin D [25(OH)D] levels, the principal circulating form of vitamin D, showed moderate declines in externalizing behavior, which includes aggression and conduct problems, over 12 months (33). This pattern of findings has also been observed in a clinical sample as decreased vitamin D was associated with increased aggression in females with schizophrenia (34). Longitudinally, vitamin D-deficient children with 25(OH)D levels below 50 nmol/L exhibited more aggressive behavior and a 1.8 times greater prevalence of clinical externalizing problems in adolescence, a median of 6 years later (35). Even at a higher threshold, initial evidence indicates that 25(OH)D levels below 75 nmol/L, reflecting vitamin D insufficiency, is associated with more aggressive behavior, particularly for reactive as opposed to proactive aggression (36). Despite these findings, three studies report a non-significant relationship between vitamin D and aggression (8, 37, 38).

The role of maternal vitamin D status on child aggression has also been investigated as vitamin D is transported from mother to fetus in the form of 25(OH)D. Children of women with high serum 25(OH)D levels (>50.7 nmol/L) at their first prenatal visit had fewer externalizing behavior symptoms at age 4 years, compared to children of women with lower 25(OH)D (39). Findings that maternal vitamin D status at other stages in pregnancy were not associated with externalizing behavior in their children (40–42) suggest that higher prenatal vitamin D status in early pregnancy may protect against childhood externalizing problems, while 25(OH)D concentrations in late pregnancy do not.

Experimentally, children with ADHD who received a supplementation in the form of 25 μg of vitamin D over 3 months exhibited significantly fewer parent and teacher-rated behavioral problems, such as fights with others and temper tantrums, compared with a control group (43). In contrast, another study showed that although healthy adolescents that took 38 μg of daily vitamin D supplementation over 3 months showed better performance on a difficult executive function task compared with a placebo group, no difference in aggressive behavior was observed (44). Some research has shed light on the effects of different vitamin D dosages. Infants who were randomly assigned to receive 10 μg or 30 μg of vitamin D daily from 2 weeks to 24 months of age did not show differences in externalizing behavior after the treatment (45). Notably, the odds of scoring 1.5 standard deviations or more on the externalizing domain was found to be higher for those who received 30 μg of vitamin D. This suggests that although some potential effectiveness of vitamin D supplementation has been observed, much higher vitamin D doses may not provide benefits for child behavior.

## Magnesium

Magnesium is an essential mineral that has been found in lower amounts in aggressive relative to non-aggressive girls (21). This finding is corroborated by results from a longitudinal study documenting that low dietary intake of magnesium in adolescents was significantly associated with increased externalizing behavior after adjusting for confounders (46). Additionally, data from a large cohort of adults show that lower magnesium intake was associated with hostility over 5 years, which is characterized in part by overt or repressed aggressive behavior (47). One study found that although low magnesium intake was not associated with externalizing behavior, it was related to increased callous-unemotional traits (48). Contrary to the results from the majority of studies assessing dietary magnesium intake, this pattern of findings was not observed when magnesium was measured using blood samples. A non-significant association between blood levels of magnesium and aggression was reported in a forensic sample using a conservative statistical test, although the study was also limited by a small sample size (8). Furthermore, using hair element analysis to assess levels of trace minerals, one study reported that higher magnesium levels were associated with greater aggression in preschool children (49). Thus, although research on aggression and dietary intake of magnesium appear to converge on similar conclusions, mixed findings are observed using other measures.

There is a noticeable lack of research on the role of magnesium on aggression in experimental studies. However, magnesium has been included in supplementations involving other micronutrients. For example, 6 mg/kg of magnesium and vitamin B6 taken daily for at least 8 weeks was found to reduce aggressiveness in children with ADHD (50), while a supplementation of 6 mg/kg of magnesium per day and 50,000 IU of vitamin D per week did not (51).

## Zinc

Like magnesium, zinc is an essential trace metal. Lower zinc concentrations assessed in diets and in blood concentrations have been linked to more behavior problems, such as aggression in children (21, 52). Similar to findings in healthy individuals, psychiatric patients with a history of aggressive, assaultive, or violent behavior had significantly lower serum zinc levels compared to patients without aggression (53, 54). These observations are in line with findings that patients with schizophrenia and a criminal history had reduced serum zinc levels compared to those without a criminal record (55). Nevertheless, some mixed findings have also been found. In a population-based cohort, although a trend showing a relationship between higher zinc intake and reduced adolescent externalizing behavior was found, the association was only marginally significant (46). Reports of an absence of a statistically significant association between blood zinc levels and aggression have also been found in a small study of forensic psychiatric inpatients and in one prospective study on schoolchildren (8, 56).

Two intervention studies have been conducted to test the relationship between zinc status and aggressive behavior. In one RCT on patients with schizophrenia, reductions in risk of aggression were found in those who received 50 mg of zinc three times a day over 6 weeks compared to patients on an atypical antipsychotic regimen alone (57). Significant differences between groups were particularly observed 4 and 6 weeks after the start of the intervention. Another RCT on schoolchildren showed that a supplementation of 10 mg of zinc, 5 days a week over 6 months did not significantly reduce parent-reported aggression scores (58). However, one major methodological issue is that dietary changes were also occurring in the schools in this trial, which may have improved nutrient intake and behavior in both experimental and placebo groups. Another consideration is that zinc supplementation may be more beneficial in individuals with higher levels of aggressive behavior.

## Discussion

This mini-review provides an overview of the salient nutritional factors associated with aggression that can be considered in the composition of nutritional supplementation to treat aggressive behavior. A number of studies provide initial evidence that lower levels of omega-3 fatty acids, vitamin D, and zinc are associated with increased aggression. These findings are broadly observed in both healthy community-based and psychiatric samples. Lower dietary levels of magnesium have also been shown to be related to increased aggression, but findings on the magnesium-aggression relationship are mixed when other measures of the trace mineral are used. The largest body of evidence stems from studies on omega-3 fatty acids. Nutritional supplementations involving omega-3 show effectiveness in reducing aggressive behavior when taken for at least 6 weeks and there is preliminary empirical support for its efficacy in reducing aggression after the treatment ends. This mini-review also highlights the dearth of studies that examine the role of vitamin D, magnesium, and zinc on aggression in an experimental way. Due to the very few studies available, firm conclusions remain to be drawn.

### Future directions

There are some limitations and research areas that require attention in future investigations. First, the mechanisms by which these nutritional components are linked to aggression are not fully understood. One common thread is that omega-3 fatty acids, vitamin D, magnesium, and zinc are linked to brain structure and function (59, 60). A model has been proposed whereby insufficient levels of vitamin D, EPA, or DHA lead to dysfunctional serotonin activation and function (61), the synthesis of which also depends on the presence of magnesium and zinc (62, 63). Low serotonin levels are suggested to lead to impairments in executive function and brain development, which are risk factors for aggression. In a test of whether omega-3 plays a role in regulating aggression through brain networks involved in distress-cued executive control over behavior, one study using electroencephalography found that Stop-P300 response to fearful and sad facial expressions mediated the relationship between lower EPA intake and increased physical aggression (64). In line with these findings, experimental studies provide evidence that supplementations of fatty fish and vitamin D can improve executive functions (44, 65). However, formal mediation tests are needed to better elucidate the mechanisms of action.

Second, one perspective that warrants further attention is that nutritional factors may help improve our understanding of the relationship between social environmental factors and aggressive behavior. For example, individuals with adverse social experiences are at an elevated risk for antisocial behavior (66). As social adversity can disrupt brain development and neuronal functioning that are relevant for the regulation of behavior, having an optimal level of nutrients that regulate brain development and functions may help counter the negative effects of social adversity. Based on this notion, one approach is to test nutritional factors as moderators of the relationship between social risk and aggression. Some evidence indicates that having sufficient 25(OH)D levels ≥30 ng/mL nullified the effect of social adversity on aggressive behavior (36), providing support for a buffering protective effect of higher vitamin D status against aggression. The nutritional factors can also help shed light on why individuals with social risk engage in more aggression. For example, given the finding that higher county crime rates were associated with lower 25(OH)D levels (67), nutritional deficits may be one pathway by which neighborhood disorder increases risk for aggressive behavior. One study documented that magnesium levels mediated the relationship between greater maternal hardship following a natural disaster and boys’ externalizing behavior at age 4 years (68).

Third, the interrelationship between nutritional factors requires consideration. Micronutrient levels, such as that of zinc and iron, affect each other. For instance, Liu et al.’s (52) study showed that combined low zinc and iron levels, but not iron alone, were significantly associated with increased reports of total behavior problems. Excessive intake of omega-6 linoleic acid may also inhibit the synthesis of omega-3 α-linolenic acid to EPA and DHA, highlighting a need to ensure a good dietary balance of omega-3 and omega-6 fatty acids (69).

Lastly, our knowledge of the nutrition-aggression link could be enhanced by considering individual differences in the sensitivity and requirement of nutrients. While most omega-3 fatty acids come from diet, variability in omega-3 levels is also determined by genetic factors (70). It has been documented that genes involved in generating and modifying omega-3 levels were associated with externalizing behavior (71). Additionally, nutrient biomarkers taken prior to a nutritional intervention, higher levels of aggression pre- treatment, and being in a higher BMI percentile were associated with greater reductions in aggression following a broad-spectrum micronutrient treatment (72). Thus, pre-treatment variables may assist in determining treatment responses to nutritional supplementation. In forensic psychiatric settings, it has been suggested that studies on nutritional supplementation consider the use of psychotropic medications. Recent trials on psychiatric inpatients and individuals with intellectual disabilities have proposed that the lack of beneficial effects from nutritional supplementation may be attributable to a possible ceiling effect due to the existing use of antipsychotics in most patients (16, 73).

## Conclusion

In summary, although this review is limited by the number of studies on nutritional factors and aggression, the available body of research shows evidence of a possible role of omega-3 fatty acids, vitamin D, magnesium intake, and zinc on aggression. They represent modifiable factors and can serve as opportunities for intervention in conjunction with other approaches to reduce aggression. This is especially as changes can be made in a non-invasive way. Omega-3 can be obtained from seafood, meat, and eggs (74). Magnesium levels are high in foods such as unrefined grains and nuts (75), while zinc is found in foods of animal origin, including dairy products (76). Nutrient deficiency is also treated through supplements. For example, as few foods contain vitamin D, supplementation has been recommended to be the best way to get additional vitamin D (77, 78). Additional experimental trials are needed to further elucidate implementation issues, such as the dosage and duration of supplementation required to elicit behavioral changes. The examination of nutritional factors also has the potential to improve our mechanistic understanding of the link between social factors and aggressive behavior.

## Author contributions

The author confirms being the sole contributor of this work and has approved it for publication.

## Conflict of interest

The author declares that the research was conducted in the absence of any commercial or financial relationships that could be construed as a potential conflict of interest.

## Publisher’s note

All claims expressed in this article are solely those of the authors and do not necessarily represent those of their affiliated organizations, or those of the publisher, the editors and the reviewers. Any product that may be evaluated in this article, or claim that may be made by its manufacturer, is not guaranteed or endorsed by the publisher.
